# The effect of surgery on the outcome of treatment for multidrug-resistant tuberculosis: a systematic review and meta-analysis

**DOI:** 10.1186/s12879-016-1585-0

**Published:** 2016-06-10

**Authors:** Rebecca C. Harris, Mishal S. Khan, Laura J. Martin, Victoria Allen, David A. J. Moore, Katherine Fielding, Louis Grandjean, the LSHTM MDR-TB surgery systematic review group, Ali Amini, Ruaridh Buchanan, Maria Krutikov

**Affiliations:** TB Centre, London School of Hygiene & Tropical Medicine, Keppel Street, London, WC1E 7HT UK; Saw Swee Hock School of Public Health, National University of Singapore, Singapore, 119077 Singapore; Royal Brompton and Harefield NHS Foundation Trust, Sydney Street, London, SW3 6NP UK; Chelsea and Westminster Hospital, 369 Fulham Road, London, SW10 9NH UK; The School of Public Health, University of the Witwatersrand, Johannesburg, South Africa; Department of Infection, Immunology and Rheumatology, Institute of Child Health, University College London, Guilford Street, London, WC1E 6BT UK

**Keywords:** Multi-drug resistant, Extensively drug resistant, Tuberculosis, Surgery, Pneumonectomy, Meta-analysis, Systematic review

## Abstract

**Background:**

In 2014 only 50 % of multidrug-resistant tuberculosis (MDR-TB) patients achieved a successful treatment outcome. With limited options for medical treatment, surgery has re-emerged as an adjuvant therapeutic strategy. We conducted a systematic review and meta-analysis to assess the evidence for the effect of surgery as an adjunct to chemotherapy on outcomes of adults treated for MDR-TB.

**Methods:**

Databases and grey literature sources were searched using terms incorporating surgery and MDR-TB. No language or publication type limits were applied. Articles published pre-1990, without a comparator group, or reporting <10 surgical participants were excluded. Two-stage sifting in duplicate was employed. Data on WHO-defined treatment outcomes were abstracted into a standardised database. Study-level risk of bias was evaluated using standardised tools. Outcome-level evidence quality was assessed using GRADE. Forest plots were generated, random effects meta-analysis conducted, and heterogeneity assessed using the I^2^ statistic.

**Results:**

Of 1024 unique citations identified, 62 were selected for full-text review and 15 retained for inclusion. A further four articles were included after bibliography/citation searching, and one additional unpublished manuscript was identified, giving 20 articles for final inclusion. Six were meta-analyses/systematic reviews and 14 were primary research articles (observational studies).

From the 14 primary research articles, a successful outcome (cured/treatment completed) was reported for 81.9 % (371/453) and 59.7 % (1197/2006) in the surgical and non-surgical group respectively, giving a summary odds ratio of 2.62 (95 % confidence interval 1.94–3.54). Loss to follow-up and treatment failure were lower in the surgery group (both *p* = 0.01). Overall GRADE quality of evidence for all outcomes considered was “very low”.

**Conclusions:**

This meta-analysis suggests that surgery as an adjunct to chemotherapy is associated with improved treatment outcomes in MDR-TB patients. However, inherent limitations in observational study design, insufficient reporting, and lack of adjustment for confounders, led to grading of the evidence as very low quality. Data on rationale for surgical referral, subsequent outcomes and resource-limited settings are scarce, precluding evidence-based recommendations on the suitability of surgery by patient characteristics or setting. It is hoped that highlighted methodological and reporting gaps will encourage improved design and reporting of future surgical studies for MDR-TB.

**Electronic supplementary material:**

The online version of this article (doi:10.1186/s12879-016-1585-0) contains supplementary material, which is available to authorized users.

## Background

It is estimated that 20 % of previously treated TB cases and 3.3 % of new TB cases worldwide have multidrug-resistant tuberculosis (MDR-TB), which is caused by bacterial strains resistant to both the two major anti-tuberculosis drugs, isoniazid and rifampicin [1]. In 2014, there were an estimated 480,000 incident cases of MDR-TB and 190,000 people died of MDR-TB [1]. Extensively drug-resistant TB (XDR-TB) is defined as MDR-TB with additional resistance to any fluoroquinolone, and to any of the three second-line injectables (amikacin, capreomycin, kanamycin). Individuals with XDR-TB have been reported by 105 countries to-date, and are estimated to account for 9.7 % of those with MDR-TB [1].

Treatment for MDR- and XDR-TB currently entails therapeutic regimens with much lower efficacy and much greater toxicity than those used for drug-susceptible TB. Recommended treatment requires at least 20 months of therapy and in 2014 only 50 % of MDR-TB patients globally had a successful treatment outcome compared to 86 % for newly diagnosed drug susceptible disease. Even with the discovery of bedaquiline and delamanid, the first anti-tuberculosis drugs with new mechanisms of action to be approved in over 40 years and the first drugs to be introduced specifically for MDR-TB combination therapy [2–4], access is limited and regimen effectiveness still remains below that of drug susceptible disease [4, 5]. Increasing drug resistance further limits the treatment options available to MDR-TB patients.

In the pre-chemotherapeutic era surgical procedures were commonly used for management of tuberculosis. Collapsing the lung by creation of an artificial pneumothorax or by plombage was regarded as an effective way to deal with lobes of affected, non-functioning lung. With the advent of effective chemotherapy however it soon became clear that medical therapy offered a superior option and enthusiasm for surgical approaches waned. In the current context of increasing drug resistant TB with far less effective medical therapy there has been an understandable resurgence of interest in the use of surgery as an adjuvant therapeutic strategy. In contrast to earlier techniques, the dominant procedures in the 21^st^ century are resection of segments, lobes or whole lungs, with collapse therapy much less used. Surgical resection can debulk disease, reducing bacillary load, and removing devitalised lung that acts as a sanctuary site for resistant organisms, poorly penetrated by drug therapies. However, removal of lung tissue reduces pulmonary capacity and thus it is crucial that pre-operative assessment takes account of the residual lung function with which the patient will be left post-operatively. Appropriate timing of surgery, before too much of the remaining lung is affected by disease, and selection of the procedure to maximize removal of non-functioning tissue whilst minimizing removal of non-diseased lung are key determinants of a successful surgical outcome.

Current treatment guidelines issued by the World Health Organization (WHO) and US Centres for Disease Control and Prevention suggest that surgical interventions may be appropriate as an adjunct to chemotherapy when skilled thoracic surgeons and good postoperative care are available [6, 7]. Intensive chemotherapy prior to surgery and postoperative chemotherapy for 12–24 months is also recommended [6, 7]. Iseman et al. established criteria for surgical intervention in MDR-TB [8], including (i) drug resistance so extensive that there is a high probability of failure or relapse, (ii) disease sufficiently localised that the majority of the disease can be resected, with the expectation of adequate cardiopulmonary capacity post surgery and (iii) sufficient drug activity to diminish the mycobacterial burden enough to facilitate probable healing of the bronchial stump. There is a specific window of opportunity for surgery, as it is rarely an immediate choice upon MDR diagnosis, but is also not suited as a last resort rescue therapy. Other guidelines refer to surgery being used for localized disease, when drug resistance is extensive, and as an adjunct to chemotherapy after at least two months of surgery with the completion of 12–24 months chemotherapy post operatively [6]. Guidelines emphasis the importance of only offering surgery in areas where there is sufficient local surgical expertise and adequate infection control available.

Systematic reviews on the application of surgery for MDR-TB were last published in 2012 [9]; and 2013 [10]. To inform the 2015/16 revision of the WHO MDR-TB treatment guidelines an updated systematic review was required which included a widened, global search of multiple international databases.

We conducted a systematic review and aggregated-data meta-analysis to assess existing evidence for the effectiveness of surgery on the outcomes of patients with MDR-TB.

## Methods

Though the methods are summarized briefly below, the full review protocol and PRISMA checklist are available in the Additional file 1 and a summary of the study protocol is registered on the prospective register of systematic reviews (PROSPERO reference: CRD42015029501) [11]. Minor amendments were made to the original protocol where clarity was required to ensure consistent interpretation. These included searching Google Scholar rather than Google and the exclusion of articles with fewer than 10 patients recruited in the surgery arm rather than overall.

### Search strategy

A comprehensive search strategy based on the PICOT framework was developed in consultation with WHO technical experts and following standard PRISMA guidelines [12] (see Additional file 1). The population of interest was defined as patients with microbiologically-confirmed MDR- or XDR-TB. The research question explored surgery as an adjunct to standard of care, therefore the comparator group was defined as those patients who received second-line chemotherapy including at least four drugs, and the intervention was defined as surgery in addition to this standard of care. The primary outcomes of interest, based on WHO definitions, were: cure, treatment completion, death, lost-to-follow-up, treatment failure, transfer out and relapse. Since the routine use of regimens including at least four such agents only became commonplace in the early 1990s, database searches were limited to 1^st^ January 1990 - 25^th^ September 2015 (the date of the database search).

We searched electronic health care databases, sources of evidence-based reviews, guidelines, and grey literature, using Pubmed (incorporating MEDLINE), Embase, Cochrane CENTRAL (including CDSR, DARE, and HTA database), WHO Global Index Medicus, WHO Clinical Trials Portal, the Union World Conference on Lung Health abstracts available on line from 2004 to 2014, OpenSIGLE databases and Google Scholar - in accordance with the specifications of each database. No language or publication type limits were applied. The specific search terms and Boolean operators used and information sources searched to identify relevant literature are detailed in Additional file 1: Tables S1, S2 and S3.

### Study selection and data extraction

Two-stage sifting in duplicate was employed. First, titles and abstracts of papers identified were independently screened for suitability for subsequent full text review based on the following pre-determined eligibility criteria: (i) recruitment of individuals with microbiologically confirmed multidrug-resistant or extensively drug resistant pulmonary tuberculosis, regardless of participant age, (ii) use of surgery as treatment for MDR-TB, as defined in the research PICOT, (iii) the reporting of data from a comparator group as defined above, and (iv) the reporting of one or more of the primary outcomes of interest (detailed below).

The following study designs were included: case series, case control study, cohort study, randomised controlled study, systematic review or meta-analysis. Narrative reviews not adding new data or new analysis of data to the existing body of knowledge, commentaries and mathematical modelling studies were excluded. Other exclusion criteria applied were studies with fewer than 10 participants receiving the intervention (surgery), any systematic review superseded by an updated systematic review and any study not in humans.

Potentially eligible publications identified at the title/abstract sifting stage were subsequently subjected to full text review by two investigators and those fulfilling the eligibility criteria were included for data abstraction and analysis. Data were extracted from eligible papers into a piloted, standardised database. Following methodology described by the York ‘Centre for Reviews and Dissemination Guidance for Undertaking Reviews in Health Care’, data extraction was conducted by one reviewer, and independently checked for accuracy by a second [13]. Unresolved disagreements in sifting or extraction were resolved by a third, independent reviewer. Citation scanning and bibliography searching was conducted for all included articles to identify any further eligible articles.

### Assessment of bias

Risk of bias was assessed at the study level using the Cochrane Collaboration Tool [14] for prospective cohort studies, the Downs and Black tool for retrospective cohort studies [15], and the AHRQ (Agency for Healthcare Research and Quality) tool for systematic reviews [16]. An adjustment was made to the Downs and Black tool such that power was interpreted as “reported” or “not reported” and incorporated with the “reporting” subscale. An assessment of quality of evidence for each key outcome across studies was conducted using the Grading of Recommendations Assessment, Development and Evaluation (GRADE) methodology [16, 17]. GRADE analysis was conducted by two reviewers in tandem, with a third for resolving discrepancies. Results were reported following PRISMA guidelines.

### Outcome measures

Primary outcomes of interest were as listed in the PICOT, following WHO definitions [18]. Unsuccessful outcomes included patients meeting the definitions of death, loss to follow up (previously called default), treatment failure, transfer out or relapse. The secondary outcome of interest was adverse events (AE) from MDR treatment and surgery. Outcomes were recorded as reported by each study.

### Analysis

Meta-analysis was used to combine results from studies to obtain a summary odds ratio (OR), comparing surgery versus non-surgery. The variance of the log OR was calculated using Woolf’s method, or from a transformation of the OR and 95 % confidence interval (CI) where only these statistics were reported. Random effects models were used to calculate summary ORs and the associated 95 % CIs, and Forest plots used to summarise data graphically. For studies with zero or 100 % of patients having the outcome in the surgery or non-surgery group, 0.5 was added to all cells to enable the variance of the log odds ratio to be estimated. We report a chi-square test for heterogeneity based on a fixed effect and the I^2^ statistic to quantify the amount of heterogeneity between the studies. We considered a value of I^2^ between 30 and 60 % as an indication of moderate heterogeneity, and >60 % an indicator of considerable heterogeneity [19]. Publication bias was investigated using funnel plots when at least 10 studies reported a given outcome.

All analyses were conducted in Stata version 13 (StataCorp LP, College Station, Texas).

## Results

### Sifting and study characteristics

**Fig. 1:**
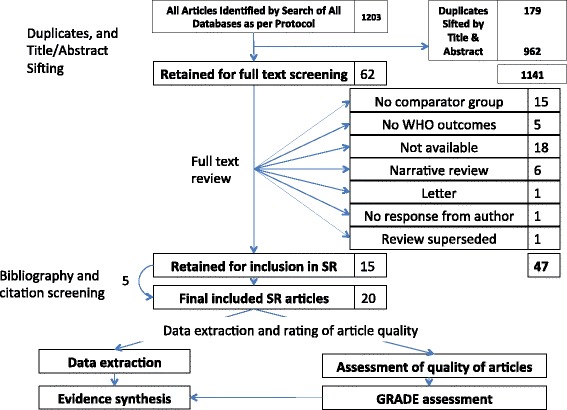
Flow chart summarising search results

A total of 1203 citation hits were retrieved from the literature search, and 1024 remained after removal of duplicates. Of these, 962 were excluded by title and abstract sifting, leaving 62 references deemed suitable for full text review. Fifteen of these were retained for inclusion and a further four articles were included after review of bibliographies in addition to one further identified unpublished manuscript, yielding a total of 20 articles for the final analysis (Fig. 1). Six included articles were meta-analyses/systematic reviews and 14 were primary research articles. Three of the six systematic reviews/meta-analyses specifically focused on the comparison of outcomes in surgery compared to medicine alone among MDR-TB patients [10, 20, 21].

Of the 14 articles presenting primary research, two and 12 were prospective and retrospective cohort studies, respectively (Table 1). The study population for the surgery group ranged from 7 to 77 patients (median 36 patients) and for the non-surgery group ranged from 41 to 1347 patients (median 162 patients). Eight articles reported prevalence of XDR-TB in the study population, which ranged from 5–100 %; 12 articles reported HIV prevalence of the study population, which ranged from 0–14 %. Only four observational studies and one meta-analysis report the numbers of study participants receiving each type of surgical procedure [21–25]. Leimane [23], Dravniece [26] and Sklyuev [27] specified the exact type of surgical intervention including lobectomy, segmentectomy, pneumonectomy or endobronchial valves, whereas the other original research articles included referred to the surgical intervention in more general terms as a resection of localised lesions. However, none of the observational studies report the outcomes of interest stratified by surgical type and therefore an aggregated meta-analysis stratified by surgery type was not possible. Only three [23, 25, 26] of the 14 primary research articles/abstracts focussed specifically on the outcomes of surgery as compared to medical treatment alone, whereas 11 articles [22, 24, 27–35] studied the outcomes of MDR-TB patients, a sub-group of which had undergone surgery.

**Table 1:** Summary of the 20 studies included in the review

Author	Year of publication	Design	Sample size overall	Sample size surgery/non surgery	Country	% Male	Age^b^	XDR	HIV
Dravniece [26]	2009	rc	254	77/177	Latvia	nr	nr	nr	nr
Gegia [35]	2012	pc	380	37/343	Georgia	nr	nr	13 %	1 %
Karagoz [34]	2009	rc	142	35/107	Turkey	100 %	39	nr	nr
Keshavjee [33]	2008	rc	608	56/552	Russia	83 %	~35	5 %	0.8 %
Kim [31]	2007	rc	211	63/148	South Korea	59 %	37	20 %	0 %
Kim [32]	2008	rc	1407	60/1347	South Korea	74 %	43	5 %	5 %
Kwak [30]	2015	rc	123	18/105	South Korea	56 %	37	21 %	0 %
Kwon [22]	2008	rc	155	35/120	South Korea	53 %	40	17 %	0 %
Leimane [23]	2005	rc	204	19/185	Latvia	77 %	43 (m); 39 (f)	nr	0.5 %
Mitnick [24]	2008	rc	48	7/41^a^	Peru	65 %	32	100 %	0 %
Shean [29]	2008	rc	491	28/463	South Africa	59 %	nr	nr	9 %
Sklyuev [27]	2013	pc	102	49/53	not described	nr	nr	nr	nr
Tahaoglu [28]	2001	rc	158	36/122	Turkey	87 %	42 (po); 36 (so)	0 %	0 %
Torun [25]	2007	rc	252	66/186	Turkey	81 %	38	nr	nr
Ahuja [36]	2012	ma	9153	499/8654	23 countries	69 %	39	0 %	14 %
Falzon [20]	2013	ma	6724	373/6351	Multiple countries	69 %	40	6 %	11 %
Fox [21]	unpublished	ma			Canada; USA; Taiwan; Korea; Japan; Estonia; UK; France	62 % (s); 70 % (ns)	37 (s); 39.4 (ns)	8.6 % (s); 5.1 % (ns)	nr
Johnston [37]	2009	ma	nr	nr	Canada	69 %	40	nr	nr
Kempker [9]	2012	sr	3218 (by calculation)	312/2906 (by calculation)	Korea, Turkey, Russia, Latvia, USA	nr	nr	nr	nr
Marrone [10]	2013	ma	5284	706/4578	Canada; USA; South Africa; Germany; Spain; Netherlands; South Korea; Turkey; Russia; Latvia; Peru; Argentina; Japan	70 %	40	Nr	nr

*rc* retrospective cohort, *pc* prospective cohort, *ma* meta-analysis, *sr* systematic review (no meta-analysis), *nr* not reported, *m* male, *f* female, *po* poor outcome, *so* successful outcome, *s* surgery, *ns* non-surgery

^a^XDR cohort only (MDR cohort has 603 patients [87 in the surgery arm and 516 in the non-surgery arm], outcomes not described)

^b^Either mean or median, unless stated

Five studies conducted meta-analyses [10, 20, 21, 36, 37] (the sixth was a systematic review without meta-analysis [9]), with three including an individualised patient data analysis [20, 21, 36]. All included studies were observational. The total number of pooled subjects included in the analyses ranged from 4238 to 9153, and the proportion of these subjects undergoing surgery was 5.5 to 13.4 %. Reported ORs from pooled data for the effect of surgery on cure or successful treatment in these meta-analyses ranged from 1.5 (95%CI 0.9–2.6) [20] to 2.24 (95 % CI 1.68–2.97) [10].

### Study outcomes

**Fig. 2:**
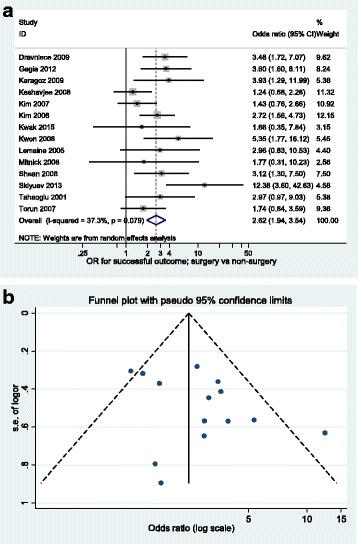
**a** Forest plot for successful outcome using random effects meta-analysis (*n* = 14 studies). **b** Funnel plot for successful outcome using random effects meta-analysis (*n* = 14 studies)

**Fig. 3:**
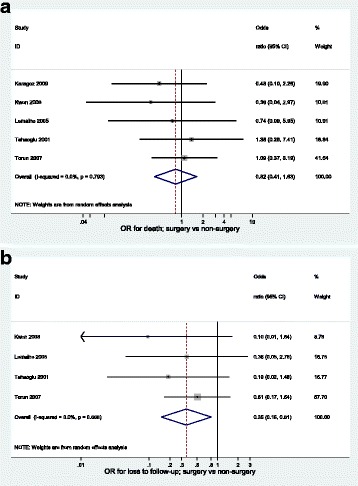
**a** Forest plot for outcome of death using random effects meta-analysis (*n* = 5 studies). **b** Forest plot for outcome of loss to follow-up using random effects meta-analysis (*n* = 4 studies)

Results of the meta-analyses are presented in Table 2. All 14 primary research articles contributed to the WHO-defined successful outcome of cured or completed treatment; 81.9 % (371/453) and 59.7 % (1197/2006) had successful outcomes in the surgery and non-surgery group, respectively. The summary OR comparing surgery versus non-surgery was 2.62 (95 % CI 1.94–3.54) [Fig. 2a]. There was weak evidence for heterogeneity (*p* = 0.08) and the I^2^ statistic was 37.3 % indicating moderate heterogeneity. There was potential overlap in patients contributing to two papers [31, 32] and so a sensitivity analysis was conducted excluding the earlier paper [31]; the resulting summary OR was 2.81 (95 % CI 2.07–3.81). Results were similar when restricted to an outcome of cure; five studies contributed to this analysis giving a summary OR of 3.03 (95 % CI 1.59–5.78) (Table 2). The summary OR for death following surgery compared to medical treatment was 0.82 (95 % CI 0.41–1.63, 5 studies), 0.35 (95 % CI 0.15–0.81, 4 studies) for loss to follow up and 0.38 (95 % CI 0.18–0.81, 5 studies) for those that failed treatment (Table 2, Fig. 3a and b). A summary OR was not calculated for the outcome of transferred out as only two studies reported data on this outcome. The funnel plot (Fig. 2b) for the analysis of successful outcome is not sufficiently asymmetrical to raise serious concerns.

**Table 2:** Summary of the meta-analyses for TB treatment outcomes

Outcome	# studies	% (n/N) surgery	% (n/N) non-surgery	Summary OR (95 % CI)	*P*-value*	*P*-value**	I^2^
Successful treatment	14 [22–35]	81.9 % (371/453)^a^	59.7 % (1197/2006)^a^	2.62 (1.94, 3.54)	<0.001	0.08	37.3 %
Successful treatment^b^	13 [22–30, 32–35]	84.1 % (328/390)^a^	59.6 % (1108/1858)^a^	2.81 (2.07, 3.81)	<0.001	0.1	30.8 %
Successful treatment^c^	17 [22–35, 38–40]	81.7 % (379/464)^a^	59.2 % (1304/2199)^a^	2.52 (1.91, 3.32)	<0.001	0.2	26.0 %
Cure	5 [22, 23, 25, 28, 34]	75.2 % (118/157)	54.9 % (308/561)	3.03 (1.59, 5.78)	0.001	0.07	54.2 %
Death	5 [22, 23, 25, 28, 34]	5.8 % (11/191)	7.2 % (52/720)	0.82 (0.41, 1.64)	0.6	0.8	0.0 %
Loss to follow-up	4 [22, 23, 25, 28]	3.8 % (6/156)	12.6 % (77/613)	0.35 (0.15, 0.81)^d^	0.01	0.7	0.0 %
Treatment failure	5 [22, 23, 25, 28, 34]	4.2 % (8/191)	11.4 % (82/720)	0.38 (0.18, 0.81)	0.01	0.99	0.0 %
Transfer out	2 [22, 23]	0 % (0/54)	2.0 % (6/350)	Not analysed			

^a^Two studies only reported OR and 95%CI and so do not contribute to the denominator and numbers of outcomes reported in the table

^b^From a sensitivity analysis which excluded one study [31] for which there may be some overlap of patients also reported in Kim et al*.* 2008 [32]

^c^From a sensitivity analysis including three studies identified in Fox et al*.* which had <10 patients in the surgery arm. For two studies [39, 40] all patients in the surgery group had a successful outcome and so 0.5 was added to all cells so that OR and 95 % CI could be calculated

^d^One study [22] had zero patients lost to follow-up in the surgery arm, so 0.5 was added to all cells so that OR and 95 % CI could be calculated

*P-value for null hypothesis OR = 1; ** *P*-value for heterogeneity

Few studies reported information pertaining to subgroups of *a priori* interest such as patients with HIV, and none reported comparative effectiveness of surgery versus a non-surgical comparator disaggregated into these subgroups. Seven [23, 28–30, 32–34] of 14 articles included in this review discussed AEs due to medication with only two of these [28, 34] providing AE data stratified by drug. Amikacin use was associated with hearing loss in 22–31 %, cycloserine use with psychosis in 3 % and with psychosis/depression in 27 % while para-amino salicylic acid was associated with gastrointestinal disturbance in 2–17 %. Four of the included studies [24, 25, 28, 34] provided a range of times at which surgery occurred after starting treatment for MDR-TB. Surgery was undertaken a median of 11.6 months after initiation of MDR treatment in the study by Mitnick et al., and a mean of 7.6 months in that by Karagoz and colleagues while surgery was undertaken after a mean of only 4.9 months in Torun et al. In the study undertaken by Tahaoglu surgery was considered after 2 months of MDR-TB treatment. Four studies [22, 24, 25, 34] considered the complications of surgical intervention which occurred in between 3 and 29 % of patients, while two studies [22, 34] specified details of surgical complications, which included surgical wound problems, empyema, bleeding, bronchopleural fistula, pneumonia, post pneumonectomy syndrome, and post-operative respiratory failure.

A sensitivity analysis was also conducted adding back in studies that were excluded from the main analysis presented here because they reported fewer than 10 patients in the surgery group; these studies were identified through having been included in earlier published meta-analyses. An additional three studies [38–40] were included in this *post-hoc* analysis for which the summary OR for successful outcome was effectively unchanged, being 2.52 (95 % CI 1.91 - 3.32) (Table 2).

### Study- and outcome- level risk of bias

The main body of available evidence was in the form of retrospective cohort studies, which scored from 7 to 19 out of 27 (median 14) when assessed for quality; this was interpreted by the authors as moderate overall quality (Table 3). Principal risks of bias arose because of little or no adjustment made for confounding [24–31, 34], unreported follow up duration or wide variations in follow up duration without reporting or adjustment for variation [23–26, 28–31], lack of outcome assessor blinding [22–35], potential indication bias [22–35], insufficient reporting of loss to follow up [28, 31], and uncertainty in population representativeness [26, 28–31, 34].

**Table 3:** Risk of bias using the Downs and Black tool^a^ for retrospective cohort studies (*n* = 12 studies)

First author, year of publication	Reporting (maximum of 11)	External validity (maximum of 3)	Internal validity, bias (maximum of 7)	Internal validity, confounding (maximum of 6)	Total (maximum of 27)
Dravniece 2009 [26]	3	0	2	2	7
Karagoz 2009 [34]	6	2	4	3	15
Keshavjee 2008 [33]	7	1	4	2	14
Kim 2007 [31]	7	0	2	1	10
Kim 2008 [32]	6	1	4	3	14
Kwak 2015 [30]	7	0	4	2	13
Leimane 2005 [23]	10	2	3	4	19
Mitnick 2008 [24]	5	2	4	2	13
Tahaoglu 2001 [28]	4	1	1	2	8
Torun 2007 [25]	8	1	3	2	14
Kwon 2008 [22]	9	2	3	4	18
Shean 2008 [29]	6	2	4	2	14

^a^Twenty-seven criteria are used to assess risk of bias. Each criterion is scored as Yes (1), No (0) or unable to determine (0). An overall score is calculated by summation, as well as four sub-scales representing “reporting” (total of 11; includes power criterion), “external validity” (total of 3), “internal validity, bias” (total of 7) and “Internal validity, confounding” (total of 6). Higher scores represent lower risk of bias

Study-level quality of prospective cohort studies was low [28, 35] due to lack of outcome assessor blinding, failure to report timing of intervention with respect to study start, and potential indication bias (Table 4).

**Table 4:** Risk of bias using the Cochrane Collaboration tool for prospective cohort studies (*n* = 2 studies)^a^

First author, year of publication	Sequence generation	Allocation concealment	Blinding of participants, personnel and outcome assessors - primary outcome	Incomplete outcome data - primary outcome	Selective outcome reporting	Other sources of bias
Gegia 2012 [35]	Unclear	Unclear	No	No	No	Yes
Sklyuev 2013 [27]	Unclear	Unclear	No	No	No	Yes

^a^ Response of “Yes” indicates that the methodological issue was appropriately addressed/managed, “No” means the issue was not managed, and “Unclear” indicates the information required to make a judgement was unclear or unavailable

Inclusion/exclusion criteria within the 14 included cohort studies were mostly broad, therefore should not have lead to significant biases in the included participants. Four of the studies did not provide any inclusion or exclusion criteria, so it is not possible to assess these for bias. One study excluded patients receiving less than three months of treatment and two excluded HIV-positive patients [22, 28, 31], which may introduce some bias or affect generalizability of the data.

Included systematic reviews [9, 10, 20, 21, 36, 37] scored positively on an average of 6 of 11 AHRQ criteria (range, 2–10) (Table 5). In general, reviews satisfactorily reported the research question, searching/sifting strategy, statistical methodology and drew conclusions supported by the data. Data extraction was almost universally inadequately described [9, 10, 20, 21, 36], only a third adequately assessed study quality [10, 37], half lacked clarity in presentation of results [9, 10, 36] and one third made no mention of declarations of either funding sources or potential conflicts of interest [10, 36].

**Table 5:** Risk of bias using the Agency for Healthcare Research and Quality tool for systematic reviews and meta-analyses (*n* = 6 studies)*

First author, year of publication	Study question	Search strategy	Inclusion and exclusion criteria	Interventions	Outcomes	Data extraction	Study quality and validity	Data synthesis and analysis	Results	Discussion	Funding or sponsorship
Marrone 2013 [10]	Yes	Yes	Yes	Partial	Partial	Partial	Yes	Yes	Partial	Yes	No
Kempker 2012 [9]	Yes	No	No	No	No	No	No	No	No	Partial	Yes
Ahuja 2012 [36]	Yes	Yes	Yes	Partial	Partial	No	Partial	Yes	Partial	Yes	Partial
Fox (unpublished) [21]	Yes	No	Yes	Yes	Yes	No	No	Yes	Yes	Yes	Yes
Falzon 2013 [20]	Yes	Yes	Yes	No	Partial	No	Partial	Yes	Yes	Yes	Yes
Johnston 2009 [37]	Yes	Yes	Yes	No	Yes	Yes	Yes	Yes	Yes	Yes	Yes

*Response of “Yes” indicates that the methodological issue was appropriately addressed/managed, “No” means the issue was not managed, and “Partial” indicates the information required to make a judgement was unclear or partially reported

Outcome level risk of bias assessed using GRADE methodology was “very low” for all primary outcomes with data available (Table 6). As all studies were observational, the maximum starting level for quality of evidence for all outcomes in the GRADE system was “low”; however, all outcomes were assessed to have serious risk of bias and therefore downgraded to “very low” quality of evidence due to indication bias and limitations with respect to management of potential confounders, representativeness of the enrolled population and variation in follow up period. In the pooled analysis, loss to follow up and treatment had strong effect sizes, but according to GRADE methodology the quality rating was not upgraded due to the existence of serious risk of bias. There was serious indirectness (defined as whether reported population, intervention and outcomes align precisely with those of interest) and imprecision (defined as when studies have wide confidence intervals around the effect estimate and when the study is insufficiently powered) in the available evidence for death, due to heterogeneity in definition (all-cause and TB-specific death), large variation in follow up time and insufficient power to detect an effect.

**Table 6:** Quality of evidence across studies for each key outcome using the Grading of Recommendations Assessment, Development and Evaluation (GRADE) methodology

**Author(s)**: Harris, R; Khan, M and Allen, V **Date**: 25/11/2015 **Question**: Surgery compared to no surgery for treatment of MDR or XDR TB **Setting**: Georgia, Latvia, Russia, South Africa, South Korea and Turkey **Bibliography**: Dravniece et al. (2009); Gegia et al. (2012); Karagoz et al. (2009); Keshavjee et al. (2008); Kim et al. (2007); Kim et al*.* (2008); Kwak et al*.* (2015); Kwon et al*.* (2008); Leimane et al*.* (2005); Mitnick et al*.* (2008); Shean et al. 2008; Sklyuev et al*.* (2013); Tahaoglu et al*.* (2001); and Torun et al*.* (2007).												
Quality assessment							№ of patients		Effect		Quality	Importance
№ of studies	Study design	Risk of bias	Inconsistency	Indirectness	Imprecision	Other considerations	Surgery	No surgery	Relative (95 % CI)	Absolute (95 % CI)		
Cured (follow up: range 0.5 to 10 years; assessed with: WHO definition)												
5	observational studies	serious ^a,b,c,d,e,f,g^	not serious^h^	not serious^i^	not serious	none^j^	118/157 (75.2 %)	308/561 (54.9 %)	OR 3.03 (1.59 to 5.78)	238 more per 1000 (from 110 more to 327 more)	⨁◯◯◯ VERY LOW	CRITICAL
Successful outcome (follow up: range 0.25 to 7 years; assessed with: Cure or treatment success, WHO definition)												
14	observational studies	serious^a,b,c,d,e,f,g,k,l,m^	not serious^n^	not serious^o^	not serious	none^j,p^	371/453 (81.9 %)^q^	1197/2006 (59.7 %)	OR 2.62 (1.94 to 3.54)^q^	198 more per 1000 (from 145 more to 243 more)	⨁◯◯◯ VERY LOW	CRITICAL
Death (follow up: range 0.5 to 10 years; assessed with: All-cause mortality or TB mortality)												
5	observational studies	serious^a,b,c,d,e,f,k,r,s,t^	not serious^n^	serious^u^	serious^t^	none^j^	11/191 (5.8 %)	52/720 (7.2 %)	OR 0.82 (0.41 to 1.64)	12 fewer per 1000 (from 41 fewer to 41 more)	⨁◯◯◯ VERY LOW	CRITICAL
Loss to follow up (previously default) (follow up: range 0.5 to 10 years; assessed with: WHO definition)												
4	observational studies	serious^a,b,c,d,e,f,v^	not serious^n^	not serious^w^	not serious	none ^j,x^	6/156 (3.8 %)	77/613 (12.6 %)	OR 0.35 (0.15 to 0.81)^y^	78 fewer per 1000 (from 21 fewer to 105 fewer)	⨁◯◯◯ VERY LOW	CRITICAL
Treatment failure (follow up: range 0.5 to 10 years; assessed with: WHO definition)												
5	observational studies	serious ^a,b,c,d,e,f,g,k^	not serious^n^	not serious^w^	not serious	none ^j,x^	8/191 (4.2 %)	82/720 (11.4 %)	OR 0.38 (0.18 to 0.81)	67 fewer per 1000 (from 20 fewer to 91 fewer)	⨁◯◯◯ VERY LOW	CRITICAL
Transfer out (follow up: Not reported)												
2	observational studies	serious ^a,b,c,f,z,aa^	not serious ^ab^	not serious	not serious^ab^	none ^aa,ac^	0/139 (0.0 %)	6/305 (2.0 %)	not estimable		⨁◯◯◯ VERY LOW	
Adverse Events from surgery (follow up: range 1.5 to 10 years)												
1	observational studies	serious ^a,b,f^	not serious ^ad^	not serious	not serious^ad^	publication bias strongly suspected ^ae^	2/66 (3 %) surgical patients died due to surgical complications.				⨁◯◯◯ VERY LOW	

*MD* mean difference, *RR* relative risk

^a^Do not address or adjust for confounders and in some studies do not fully describe population - Dravniece et al. (2009); Karagoz et al*.* (2009); Kim et al. (2007); Kwak et al. (2015); Kwon et al. (2008); Mitnick et al. (2008); Shean et al*.* (2008); Sklyuev et al*.* (2013); Tahaoglu et al. (2001) and Torun et al*.* (2007)

^b^ Retrospective observational studies do not have randomisation and have inherent bias in who is offered surgery - Dravniece et al*.* (2009); Karagoz et al. (2009); Keshavjee et al*.* (2008); Kim et al*.* (2007); Kim et al. (2008); Kwak et al*.* (2015); Kwon et al*.* (2008); Leimane et al. (2005); Mitnick et al*.* (2008); Shean et al*.* 2008; Tahaoglu et al*.* (2001) and Torun et al. (2007)

^c^ Uncertainty in representativeness of study population - Dravniece et al. (2009); Karagoz et al. (2009); Kim et al*.* (2007); Kwak et al*.* (2015); Kwon et al*.* (2008); Shean et al*.* 2008; and Tahaoglu et al*.* (2001)

^d^ No estimate of variability given - Dravniece et al*.* (2009) and Tahaoglu et al. (2001)

^e^ Number lost to follow up reported, but characteristics not described - Tahaoglu et al*.* (2001)

^f^ Length of follow up not described or adjusted for in analysis - Dravniece et al*.* (2009); Kim et al*.* (2007); Kwak et al*.* (2015); Kwon et al*.* (2008); Leimane et al. (2005); Mitnick et al. (2008); Shean et al. (2008); Tahaoglu et al*.* (2001); and Torun et al*.* (2007)

^g^ In surgical studies, it is not possible to blind patients or study team. Outcome assessors could be blinded, and is somewhat important for assessing cure using smear as an outcome. However laboratory assessment is generally conducted by different personnel than the diagnosing physician. For treatment success/failure there is a risk of reporting bias due to lack of blinding where data are programmatic, as there may be over-reporting due to programmatic targets and could be biased by knowledge of surgical status

^h^ Moderate I-squared (54.2 %) and overlapping CIs between studies so not downgraded

^i^ Some variation in duration of follow up in outcome definition, however is not downgraded as alone this is not classified as serious issue for this outcome

^j^ All studies are cohort, therefore may be some confounding due to patient allocation to surgery or no surgery. Patients who are more unwell may be more likely to be recommended for surgery (therefore causing underestimate of effect size), however the most sick are often not offered surgery as they may be too unwell or disease too disseminated to allow surgery (therefore overestimating effect size). In addition, there may be variation in the population offered surgery by setting or surgeon. As there is a specific window for surgery, these biases may have an impact on estimation of effect size, though it is unclear whether they would bias the estimation in a particular direction, and are a reflection of the reality of the patient group offered surgery. Therefore, the reviewers decided not to upgrade or downgrade the rating

^k^ Reports number, but not summary statistics or precision for this specific outcome - Leimane et al. (2005) and Mitnick et al*.* (2008)

^l^ Abstract only, outcome and patient characteristics not clearly described - Dravniece et al*.* (2009)

^m^ Loss to follow up not reported for surgical vs non-surgical patients - Kim et al*.* (2007)

^n^ Low I-squared and overlapping CIs between studies, so not downgraded

^o^ Most studies followed WHO outcome definitions. Some variation in duration of follow up to assess outcome but not downgraded as alone is not classified as serious issue for this outcome

^p^ Empty lower right quadrant of funnel plot. However, it seems that smaller (less precise) studies are reporting lower effect estimate so if publication bias were to exist this would suggest the current estimate effect measure is conservative. Per protocol, studies with <10 surgical participants were excluded, therefore the very smallest of studies were not included. Plot is not sufficiently asymmetrical to raise serious concerns, and any bias would appear to cause an underestimate of effect, therefore quality not downgraded

^q^ *n*= 13 for OR estimates, but *n *= 11 for numbers of patients summarised in the table, as 2 studies only report effect estimate rather than the number of patients with the outcome and the denominator

^r^ In surgical studies, it is not possible to blind patients or study team. Outcome assessors could be blinded, but unimportant in mortality outcome as no subjectivity in assessment

^s^ Time period of follow up very variable, and for patients with follow up for <2 years the follow up period is potentially insufficient for mortality outcome - Shean et al*.* (2008) and Torun et al*.* (2007)

^t^ Pooled CIs cross the null. Event rate is low and post-hoc optimal information size calculation indicated number included in assessment of this outcome is too low to give sufficient power

^u^ Variation between studies in outcome definition used (all-cause vs TB-only). Unclear/variable period over which death was assessed (e.g. died during treatment, within 6 months of completion, or after 2 years)

^v^ In surgical studies, it is not possible to blind patients or study team. Outcome assessors could be blinded, but where data are programmatic they are unlikely to be. This could introduce underestimate in reporting of default, but this bias is unlikely to vary between study groups

^w^ Mostly use WHO definition, minor variation in definition in some studies, but sufficiently direct not to downgrade

^x^ OR (similar to RR given infrequency of event) is <0.5 and the upper confidence interval would still provide a clinically significant benefit, therefore this would be considered a large effect size. However, the quality are not upgraded as according to GRADE methodology this should not be done if the risk of bias is serious

^y^ *N* = 2 studies had no patients lost to follow-up in the surgery group, so 0.5 has been added to all cells in order that a CI can be calculated. The summary OR restricted to the 2 studies that had at least one patient lost to follow-up in each group is 0.47 (95 % CI 0.18, 1.24)

^z^ Although reported separately, unlikely that clear differentiation has been made between LTFU and transfer out

^aa^ Suspected underreporting of outcome, but uncertain as to how this would impact the conclusions

^ab^ No pooled estimate, so insufficient evidence to assess

^ac^ Only 2 publications, so not possible to assess publication bias, but given how few report this outcome publication bias may be plausible

^ad^ One study and no comparator group so not possible to estimate

^ae^ Likely that complications occurred in other studies, but have either not been reported or have been included in all-cause deaths

## Discussion and conclusions

This meta-analysis of 14 observational studies indicates a substantially increased likelihood of a successful treatment outcome (cure or treatment completion) in MDR-TB patients undergoing adjunctive surgery (OR 2.62, 95 % CI 1.94–3.54). Restricting the outcome of interest to cure reveals a similar effect size, albeit with wider confidence intervals reflecting the smaller number of contributing studies (5 studies, OR 3.03, 95 % CI 1.59–5.78).

The direction of this positive effect of surgery on treatment success is consistent with previous reviews by Marrone et al. [10] (OR 2.24, 95 % CI 1.68–2.97) and Falzon et al*.* [20] (OR 1.5, 95%CI 0.9–2.6), albeit with a marginally greater magnitude. A strength of our review is the large number of globally-representative databases searched, therefore the meta-analysis reported here included a number of additional pre-2012 papers and also included several more recently published papers [20, 30].

Adjunctive surgery was also associated with a reduction in loss to follow-up and treatment failure. For both these analyses the estimated effects reported by individual studies all reported a reduction due to surgery. The confidence intervals of the summary estimate for effect of surgery on death (assessed as TB mortality or all cause mortality) crossed the null (OR 0.82, 95 % CI 0.41–1.64), therefore there it was not possible to demonstrate that surgery has an effect upon mortality.

Analyses were planned for several sub-populations, including children, patients with diabetes, pregnant women and patients with HIV, but either no relevant data were identified or reporting was insufficient to stratify for these subgroups. For patients with HIV, only one meta-analysis [37] that included 12 studies with data on HIV patients was identified; however, none of the studies analysed the effect of surgery stratified by HIV status. Only three of the 14 original research articles clearly stated the type of surgery undertaken, the remainder classified surgical intervention in more general terms as resective surgery of localized lesions. As the studies provided very limited, if any, information on the type of surgery, sub-analyses comparing types of surgery were not possible. Given the considerable difference between a pneumonectomy and a segmentectomy this remains an important knowledge gap and unsatisfactorily forces analyses into the binary surgery/non-surgery approach which clearly conceals major heterogeneity in procedures utilised.

As discussed recently by Roberts and Ker, the inclusion of very small studies in systematic reviews can lead to an exaggerated estimate of treatment effect due to publication bias and the tendency towards lower quality and oversight [41]. Small case series often have less rigorous inclusion criteria and are thus more prone to recruitment bias. Therefore, in this review studies reporting fewer than 10 surgical cases were excluded. A sensitivity analysis including such articles demonstrated that this had little effect on the effect estimates and did not affect the conclusions. Two papers by Kim et al. [31, 32] had potentially overlapping patients as two of the ten years reported in the 2007 paper may have also been included in the 2008 paper. A sensitivity analysis to exclude the 2007 paper gave a similar effect estimate, demonstrating that this potential overlap would not bias conclusions drawn.

Overall, the quality of evidence across each outcome measure with available data was assessed as “very low” using GRADE methodology. Most of the outcomes had clear directionality and sufficiently narrow CIs to suggest that surgery had a positive effect on patient outcomes, with the exception of death for which the summary CIs crossed the null. However, the event rate for death was low and a post-hoc optimum information size calculation indicated that there was insufficient power. This combined with the heterogeneity in the outcome definition suggest that the null result could conceivably be a product of these methodological issues. Therefore the reviewers are cautious to over-interpret the null result.

The most notable methodological issue is the absence of experimental studies in the current body of evidence, which thus consists entirely of observational studies (mainly retrospective cohort studies). Reliance on such study designs mean inherent biases such as lack of blinding or randomisation cannot be managed, and residual confounding is not managed through study design. The most important limitation in the meta-analysis was the inability to appropriately adjust for known confounding: eight of the 12 retrospective cohort studies did not sufficiently address confounding [24–26, 28–31, 34], which could have a major influence upon the reported treatment effects.

No studies explicitly evaluated the timing of surgery or considered it as a confounding variable in a multivariate regression, though two reported a wide range over which surgery was performed [28, 34]. A window of opportunity exists for indication of surgery, as those with less severe disease tend to be allocated to medical treatment alone, whereas in more severe disease there may be reticence to operate owing to the risk of death or complications during the procedure and the anticipated inadequacy of post-surgery respiratory reserve. Although the timing of surgery is highly likely to influence outcome, the available data on this important confounding variable are scarce.

The experience of the surgeon and quality of surgical facilities and post-surgical care likely affect estimation of surgical risk in different settings. This, combined with the lack of an evidence-based well-established guideline, results in variation in the criteria used to determine whether surgery is indicated. Since groups receiving surgery are heterogeneous and their characteristics not well reported, the authors propose the development of a standardised minimum dataset for use by surgeons to record their rationale for the surgery/no surgery decision (e.g. clinical characteristics of lung involvement, safety considerations), and for clearly recording the intervention and patient outcomes. Incorporated in to an international registry (potentially online) this observational data could quickly grow into an informative dataset to help understanding of international practice, the drivers of heterogeneity and practices more commonly associated with a more favourable outcome.

Studies were identified from Asia, Africa and Europe, so provide some global representativeness, but were mostly from relatively high income settings. Therefore, although surgery was largely associated with positive outcomes in the results reported here, caution is advised in generalising this finding to more resource-constrained settings. Surgical expertise is a clear determinant of outcome and most studies included in this meta-analysis were from well-resourced tertiary referral centres. Clearly such low-frequency surgery should be undertaken only in centres of expertise; whether in low income settings the rates of post-operative complications such as infection, pneumothorax or other morbidities arising from less intensive post-operative supervision would be different is not known.

The paucity of reported adverse event data for both medical and surgical arms also limit our conclusions. Previously reported morbidity from surgery for MDR-TB ranges between 9.4 to 46 % [42] while the frequent side effects occurring during 2 years of anti-tuberculosis treatment for MDR-TB are well documented [43]. Consideration of surgical complication risk is an important part of the decision-making process for surgeon and patient, though as surgery is an adjuvant not a replacement therapy, patients who undergo surgical resection are not spared any of the risk of drug therapy adverse effects to which their non-surgical counterparts are also exposed.

This work indicates that the existing evidence, determined through the GRADE assessment as being of very low quality, may support the use of adjuvant surgery in the management of MDR-TB. However, due to potentially important residual confounding, caution is advised in the interpretation of these results. It is also self evident that surgery is not a sensible proposition for all MDR-TB patients. The nature of patient and surgeon decision-making about whether to proceed to surgery is not at all well captured by this type of analysis nor by the constituent articles, and remains a key obstacle to the generation of unbiased evidence with minimal confounding.

## Abbreviations

AHRQ, Agency for Healthcare Research and Quality; CI, confidence interval; GRADE, Grading of recommendations, assessment, development and evaluations; MDR TB, multidrug-resistant tuberculosis; OR, odds ratio; XDR TB, extensively drug resistant tuberculosis.

## Additional file

Additional file 1: Table S1. Search terms applied. **Table S2.** Information sources searched to identify relevant literature. **Table S3.** Search limits applied in Pubmed. **Table S4.** PRISMA 2009 Checklist. (DOCX 24 kb)
